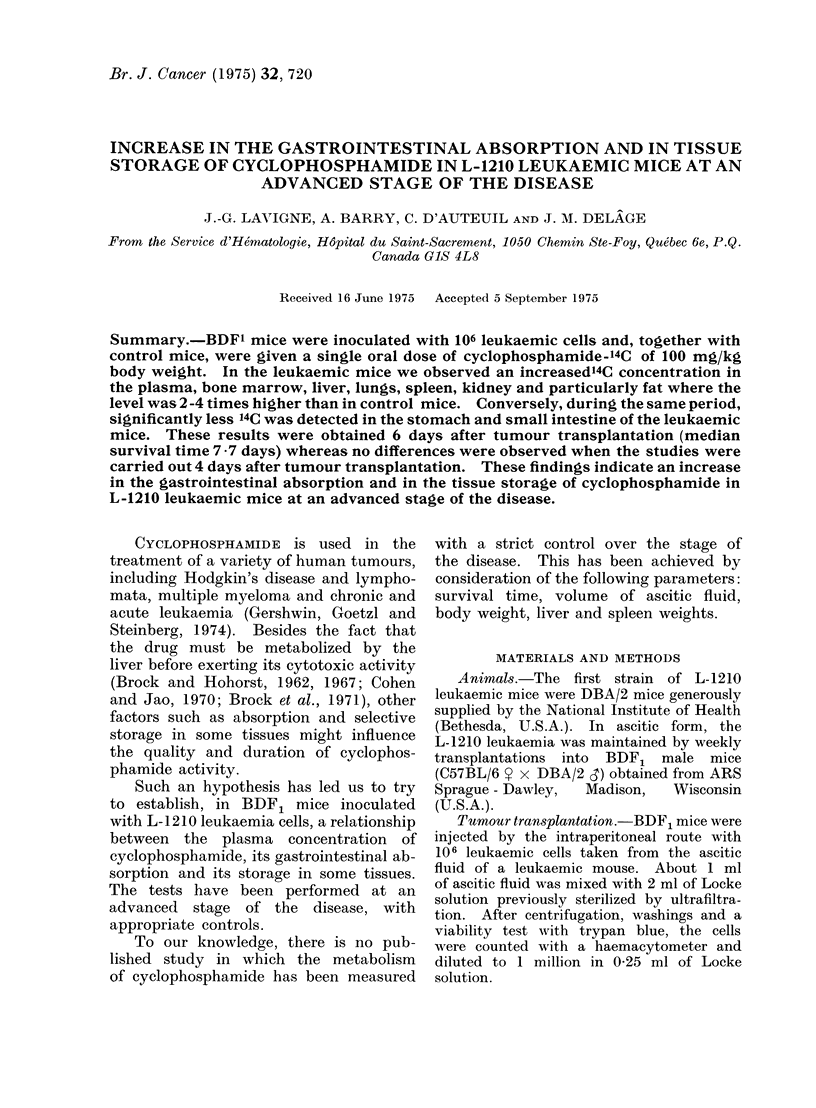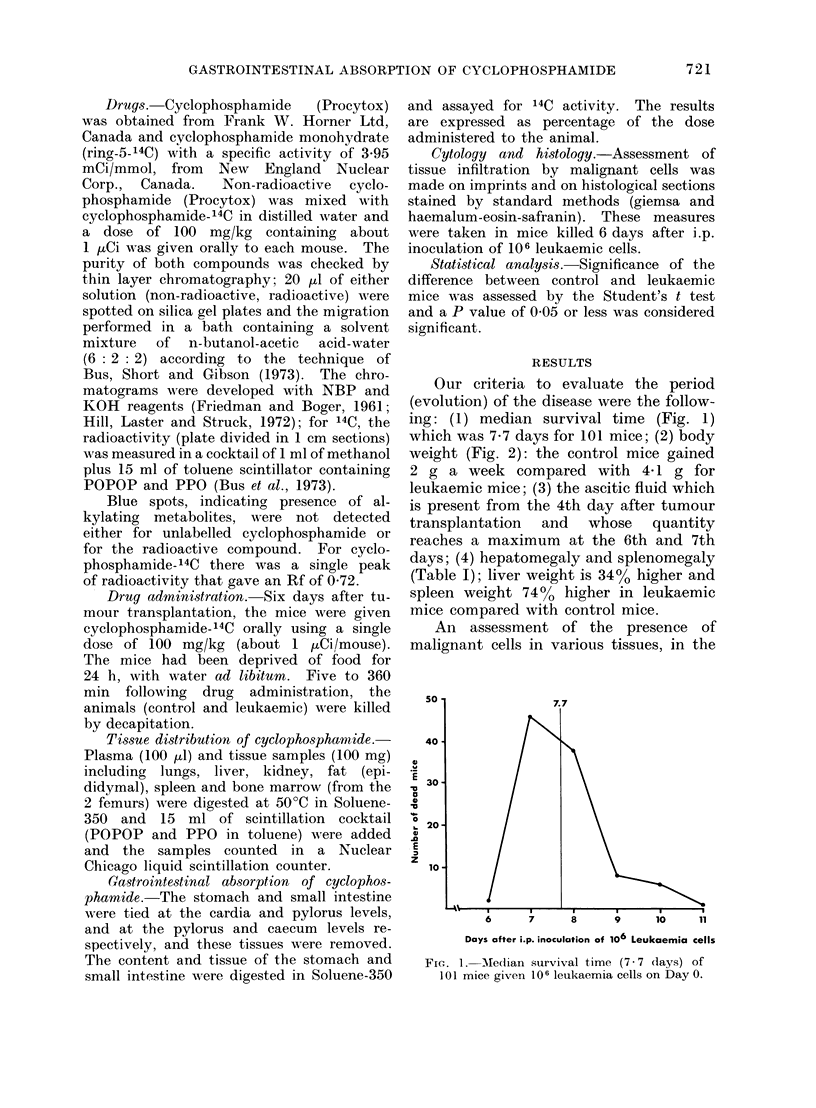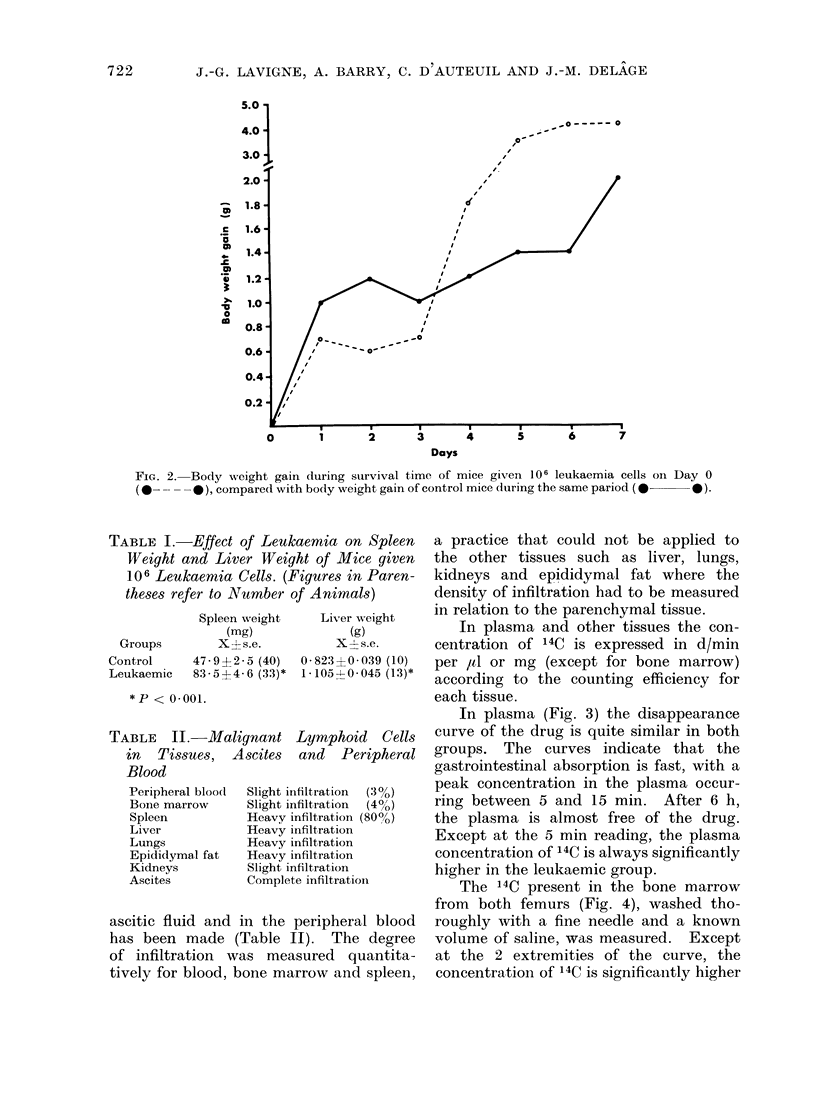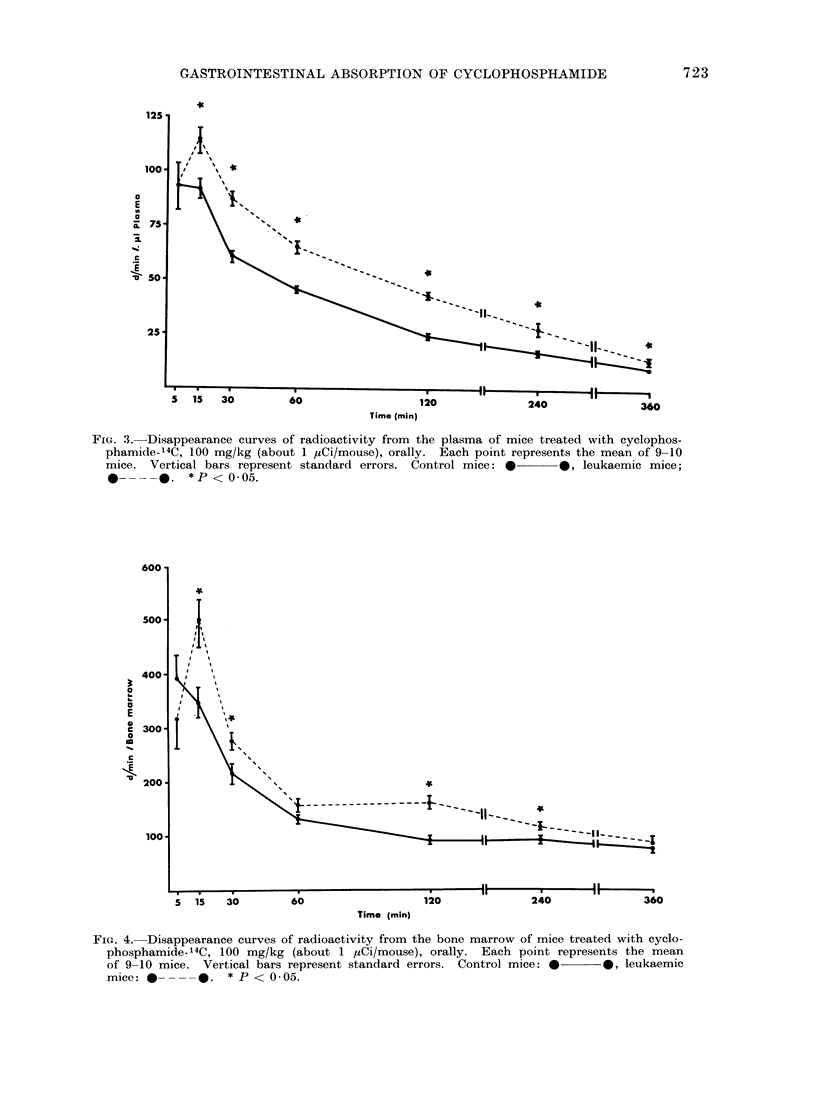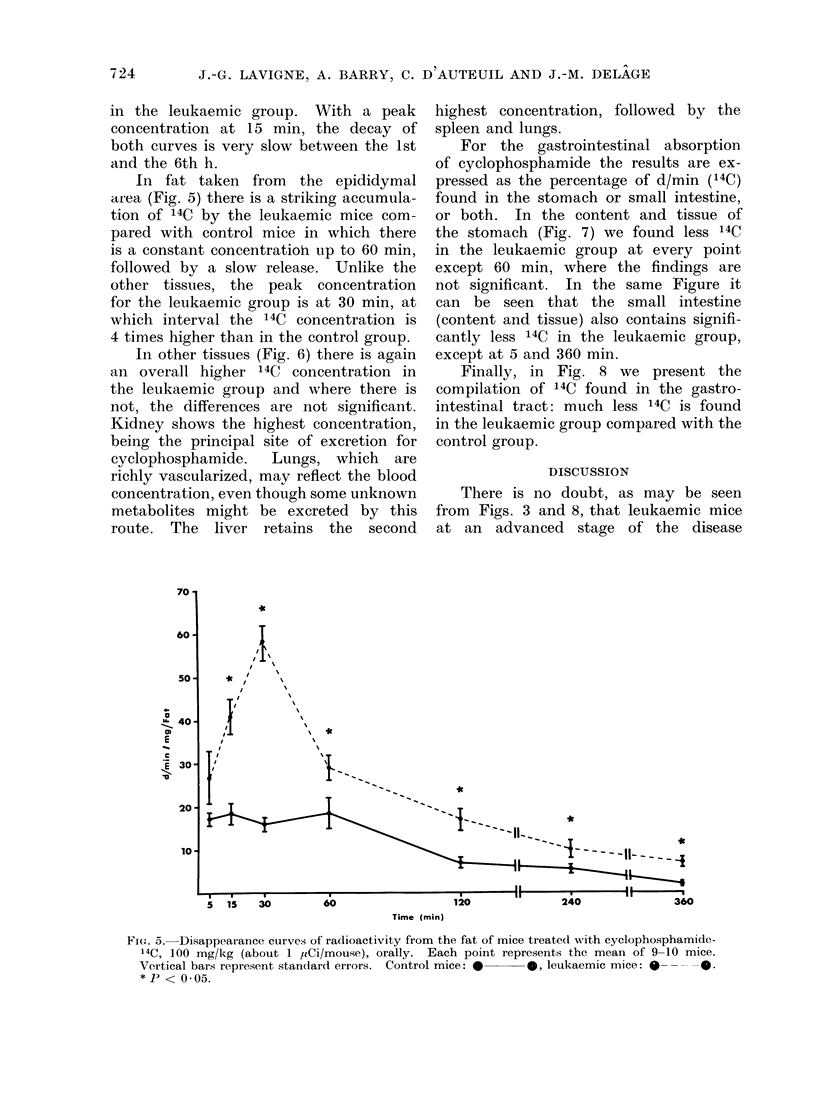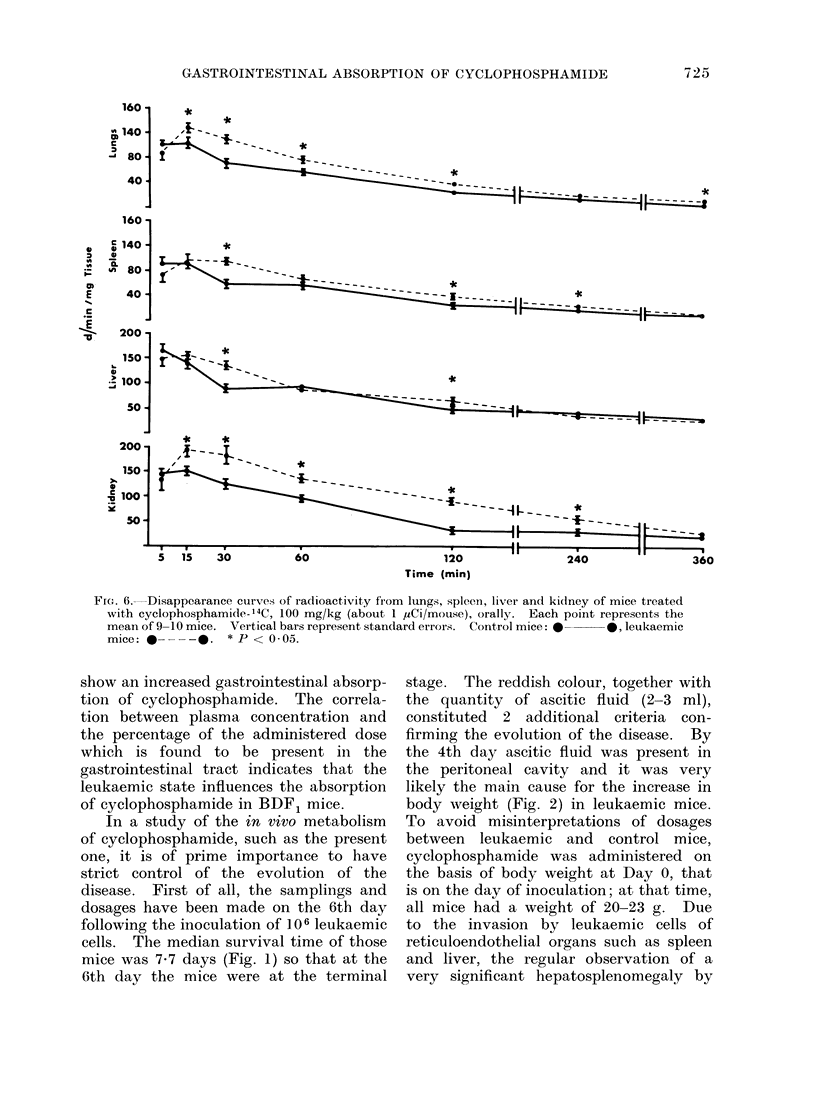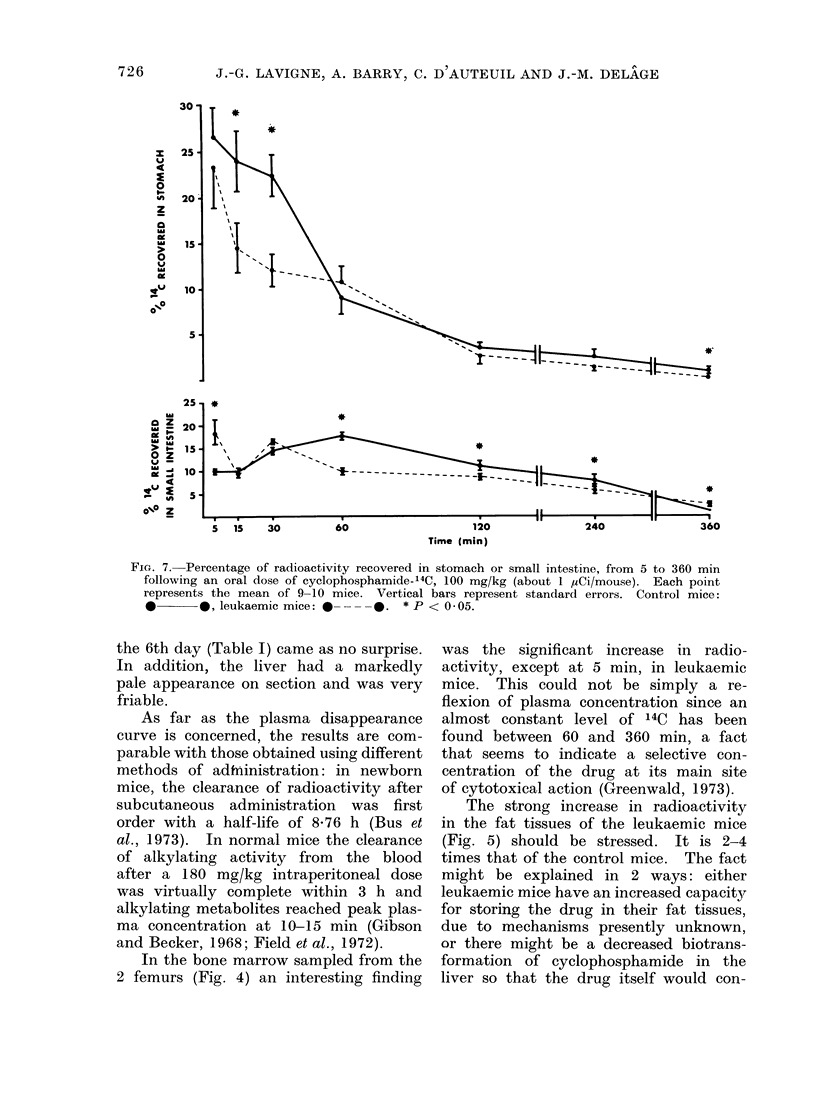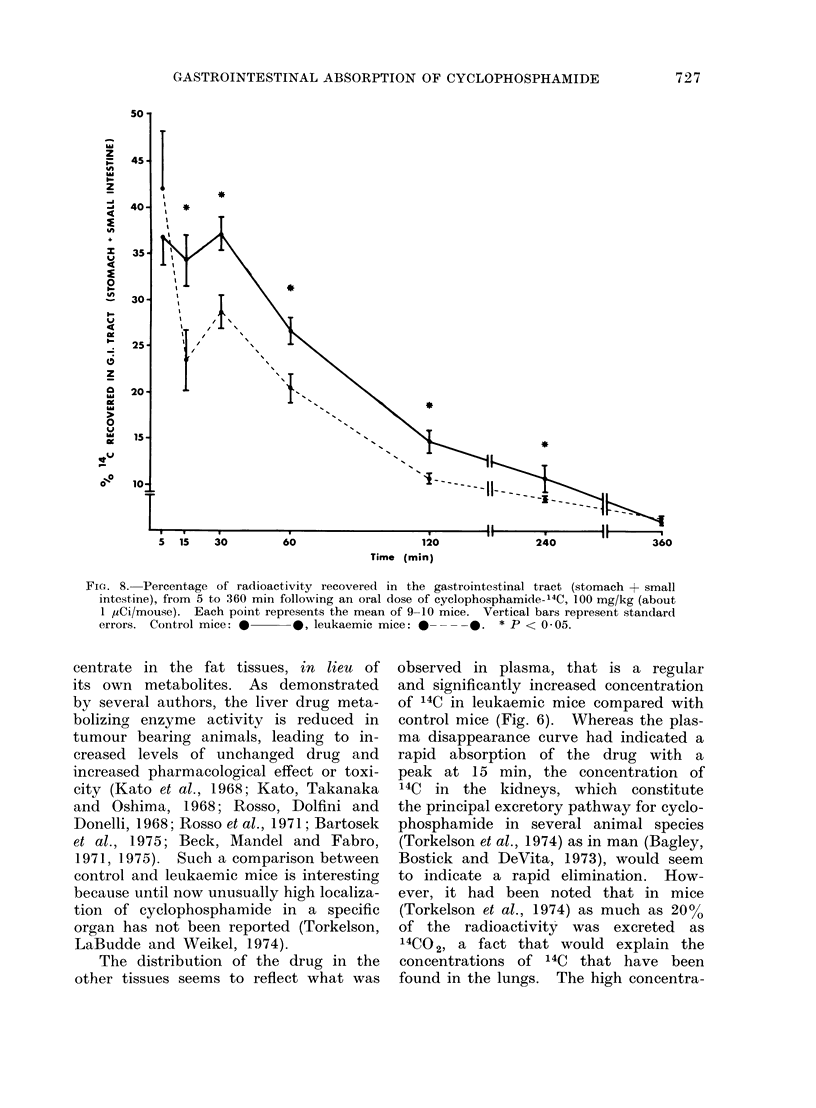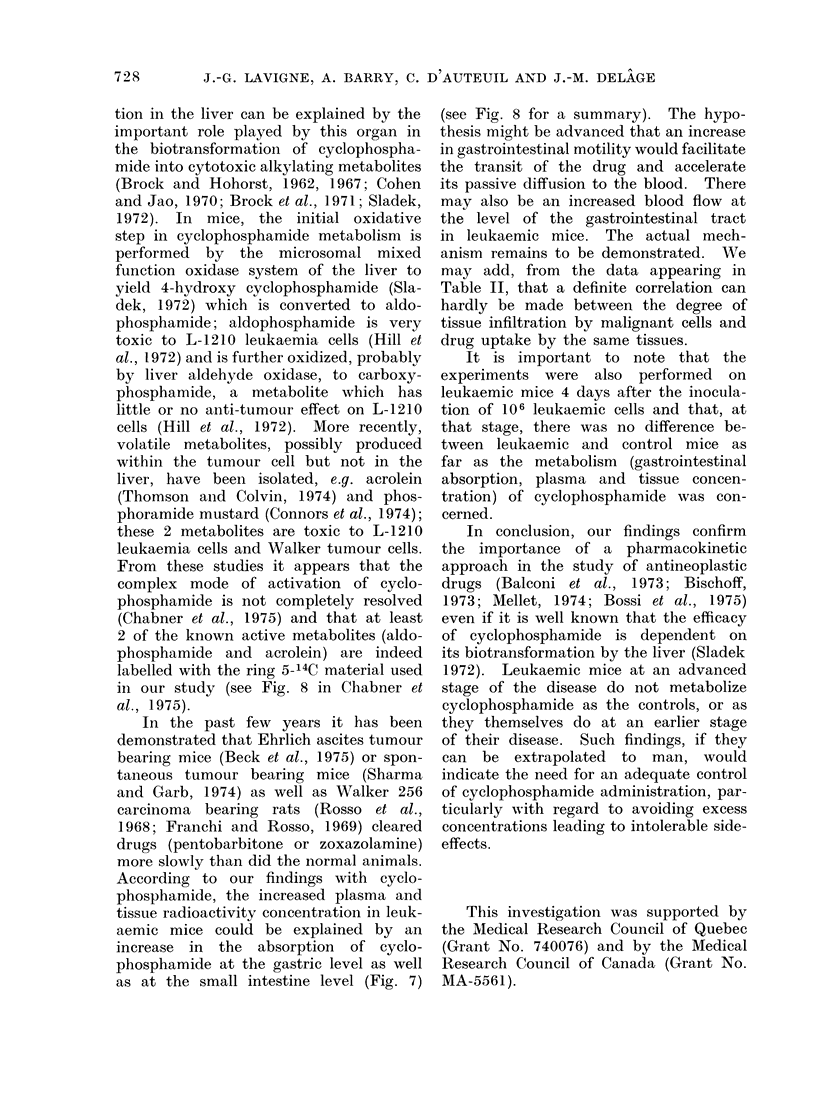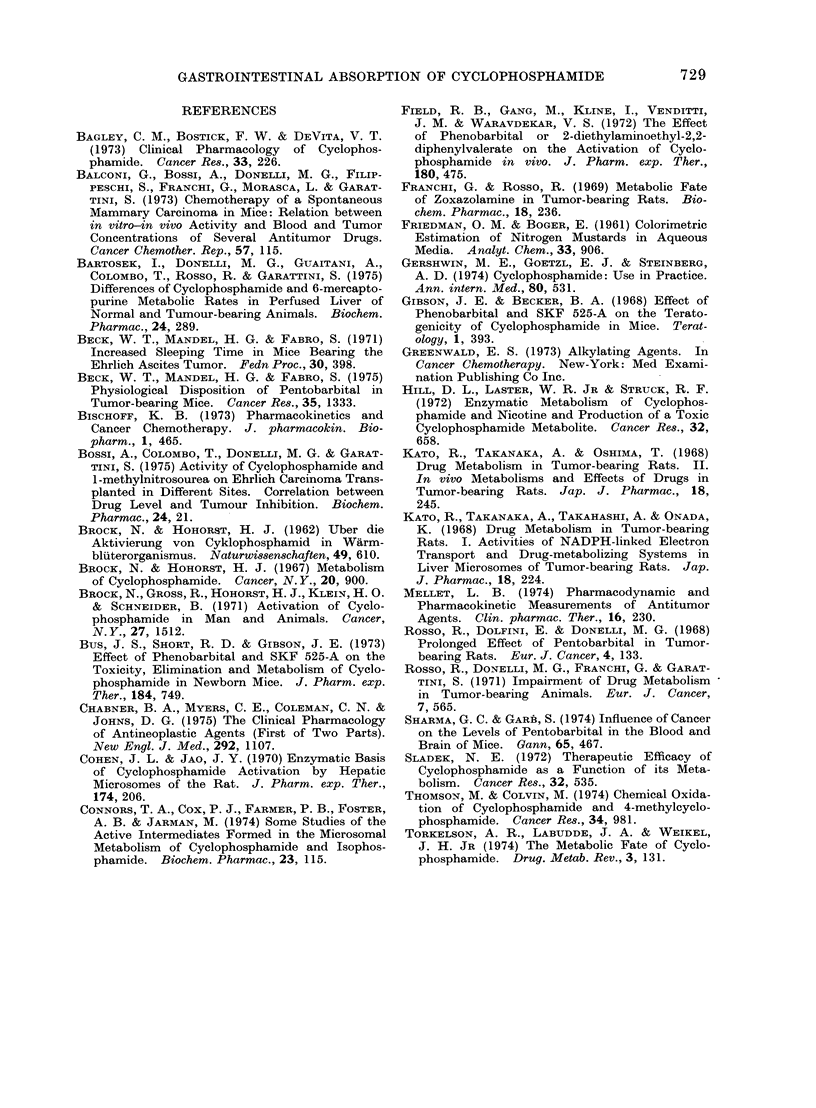# Increase in the gastrointestinal absorption and in tissue storage of cyclophosphamide in L-1210 leukaemic mice at an advanced stage of the disease.

**DOI:** 10.1038/bjc.1975.283

**Published:** 1975-12

**Authors:** J. G. Lavigne, A. Barry, C. D'Auteuil, J. M. Delâge

## Abstract

BDF1 mice were inoculated with 10(6) leukaemic cells and, together with control mice, were given a single oral dose of cyclophosphamide-14C of 100 mg/kg body weight. In the leukaemic mice we observed an increased 14C concentration in the plasma, bone marrow, liver, lungs, spleen, kidney and particularly fat where the level was 2-4 times higher than in control mice. Conversely, during the same period, significantly less 14C was detected in the stomach and small intestine of the leukaemic mice. These results were obtained 6 days after tumour transplantation (median survival time 7.7 days) whereas no differences were observed when the studies were carried out 4 days after tumour transplantation. These findings indicate an increase in the gastrointestinal absorption and in the tissue storage fo cyclophosphamide in L-1210 leukaemic mice at an advanced stage of the disease.


*Br. J. Cancer* (1975) **32**, 720

### INCREASE IN THE GASTROINTESTINAL ABSORPTION AND IN TISSUE STORAGE OF CYCLOPHOSPHAMIDE IN L-1210 LEUKAEMIC MICE AT AN ADVANCED STAGE OF THE DISEASE

J.-G. LAVIGNE, A. BARRY, C. D'AUTEUIL AND J. M. DELÂGE

*From the Service d'Hématologie, Hôpital du Saint-Sacrement, 1050 Chemin Ste-Foy, Québec 6e, P.Q. Canada G1S 4L8*

Received 16 June 1975 Accepted 5 September 1975

**Summary.—BDF[1] mice were inoculated with $10^6$ leukaemic cells and, together with control mice, were given a single oral dose of cyclophosphamide-$^{14}C$ of 100 mg/kg body weight. In the leukaemic mice we observed an increased $^{14}C$ concentration in the plasma, bone marrow, liver, lungs, spleen, kidney and particularly fat where the level was 2-4 times higher than in control mice. Conversely, during the same period, significantly less $^{14}C$ was detected in the stomach and small intestine of the leukaemic mice. These results were obtained 6 days after tumour transplantation (median survival time 7·7 days) whereas no differences were observed when the studies were carried out 4 days after tumour transplantation. These findings indicate an increase in the gastrointestinal absorption and in the tissue storage of cyclophosphamide in L-1210 leukaemic mice at an advanced stage of the disease.**

CYCLOPHOSPHAMIDE is used in the treatment of a variety of human tumours, including Hodgkin's disease and lymphomata, multiple myeloma and chronic and acute leukaemia (Gershwin, Goetzl and Steinberg, 1974). Besides the fact that the drug must be metabolized by the liver before exerting its cytotoxic activity (Brock and Hohorst, 1962, 1967; Cohen and Jao, 1970; Brock *et al.*, 1971), other factors such as absorption and selective storage in some tissues might influence the quality and duration of cyclophosphamide activity.

Such an hypothesis has led us to try to establish, in $BDF_1$ mice inoculated with L-1210 leukaemia cells, a relationship between the plasma concentration of cyclophosphamide, its gastrointestinal absorption and its storage in some tissues. The tests have been performed at an advanced stage of the disease, with appropriate controls.

To our knowledge, there is no published study in which the metabolism of cyclophosphamide has been measured with a strict control over the stage of the disease. This has been achieved by consideration of the following parameters: survival time, volume of ascitic fluid, body weight, liver and spleen weights.

### MATERIALS AND METHODS

*Animals.*—The first strain of L-1210 leukaemic mice were DBA/2 mice generously supplied by the National Institute of Health (Bethesda, U.S.A.). In ascitic form, the L-1210 leukaemia was maintained by weekly transplantations into $BDF_1$ male mice (C57BL/6 ♀ × DBA/2 ♂) obtained from ARS Sprague - Dawley, Madison, Wisconsin (U.S.A.).

*Tumour transplantation.*—$BDF_1$ mice were injected by the intraperitoneal route with $10^6$ leukaemic cells taken from the ascitic fluid of a leukaemic mouse. About 1 ml of ascitic fluid was mixed with 2 ml of Locke solution previously sterilized by ultrafiltration. After centrifugation, washings and a viability test with trypan blue, the cells were counted with a haemacytometer and diluted to 1 million in 0·25 ml of Locke solution.

*Drugs.*—Cyclophosphamide (Procytox) was obtained from Frank W. Horner Ltd, Canada and cyclophosphamide monohydrate (ring-5-$^{14}C$) with a specific activity of 3·95 mCi/mmol, from New England Nuclear Corp., Canada. Non-radioactive cyclophosphamide (Procytox) was mixed with cyclophosphamide-$^{14}C$ in distilled water and a dose of 100 mg/kg containing about 1 $\mu$Ci was given orally to each mouse. The purity of both compounds was checked by thin layer chromatography; 20 $\mu$l of either solution (non-radioactive, radioactive) were spotted on silica gel plates and the migration performed in a bath containing a solvent mixture of n-butanol-acetic acid-water (6 : 2 : 2) according to the technique of Bus, Short and Gibson (1973). The chromatograms were developed with NBP and KOH reagents (Friedman and Boger, 1961; Hill, Laster and Struck, 1972); for $^{14}C$, the radioactivity (plate divided in 1 cm sections) was measured in a cocktail of 1 ml of methanol plus 15 ml of toluene scintillator containing POPOP and PPO (Bus *et al.*, 1973).

Blue spots, indicating presence of alkylating metabolites, were not detected either for unlabelled cyclophosphamide or for the radioactive compound. For cyclophosphamide-$^{14}C$ there was a single peak of radioactivity that gave an Rf of 0·72.

*Drug administration.*—Six days after tumour transplantation, the mice were given cyclophosphamide-$^{14}C$ orally using a single dose of 100 mg/kg (about 1 $\mu$Ci/mouse). The mice had been deprived of food for 24 h, with water *ad libitum.* Five to 360 min following drug administration, the animals (control and leukaemic) were killed by decapitation.

*Tissue distribution of cyclophosphamide.*—Plasma (100 $\mu$l) and tissue samples (100 mg) including lungs, liver, kidney, fat (epididymal), spleen and bone marrow (from the 2 femurs) were digested at 50°C in Soluene-350 and 15 ml of scintillation cocktail (POPOP and PPO in toluene) were added and the samples counted in a Nuclear Chicago liquid scintillation counter.

*Gastrointestinal absorption of cyclophosphamide.*—The stomach and small intestine were tied at the cardia and pylorus levels, and at the pylorus and caecum levels respectively, and these tissues were removed. The content and tissue of the stomach and small intestine were digested in Soluene-350 and assayed for $^{14}C$ activity. The results are expressed as percentage of the dose administered to the animal.

*Cytology and histology.*—Assessment of tissue infiltration by malignant cells was made on imprints and on histological sections stained by standard methods (giemsa and haemalum-eosin-safranin). These measures were taken in mice killed 6 days after i.p. inoculation of $10^6$ leukaemic cells.

*Statistical analysis.*—Significance of the difference between control and leukaemic mice was assessed by the Student's $t$ test and a $P$ value of 0·05 or less was considered significant.

### RESULTS

Our criteria to evaluate the period (evolution) of the disease were the following: (1) median survival time (Fig. 1) which was 7·7 days for 101 mice; (2) body weight (Fig. 2): the control mice gained 2 g a week compared with 4·1 g for leukaemic mice; (3) the ascitic fluid which is present from the 4th day after tumour transplantation and whose quantity reaches a maximum at the 6th and 7th days; (4) hepatomegaly and splenomegaly (Table I); liver weight is 34% higher and spleen weight 74% higher in leukaemic mice compared with control mice.

An assessment of the presence of malignant cells in various tissues, in the

FIG. 1.—Median survival time (7·7 days) of 101 mice given $10^6$ leukaemia cells on Day 0.

FIG. 2.—Body weight gain during survival time of mice given $10^6$ leukaemia cells on Day 0 (●– – – –●), compared with body weight gain of control mice during the same pariod (●———●).

TABLE I.—*Effect of Leukaemia on Spleen Weight and Liver Weight of Mice given $10^6$ Leukaemia Cells. (Figures in Parentheses refer to Number of Animals)*

| Groups | Spleen weight (mg) X±s.e. | Liver weight (g) X±s.e. |
|---|---|---|
| Control | 47·9±2·5 (40) | 0·823±0·039 (10) |
| Leukaemic | 83·5±4·6 (33)* | 1·105±0·045 (13)* |

$^*P < 0{\cdot}001$.

TABLE II.—*Malignant Lymphoid Cells in Tissues, Ascites and Peripheral Blood*

| | |
|---|---|
| Peripheral blood | Slight infiltration (3%) |
| Bone marrow | Slight infiltration (4%) |
| Spleen | Heavy infiltration (80%) |
| Liver | Heavy infiltration |
| Lungs | Heavy infiltration |
| Epididymal fat | Heavy infiltration |
| Kidneys | Slight infiltration |
| Ascites | Complete infiltration |

ascitic fluid and in the peripheral blood has been made (Table II). The degree of infiltration was measured quantitatively for blood, bone marrow and spleen, a practice that could not be applied to the other tissues such as liver, lungs, kidneys and epididymal fat where the density of infiltration had to be measured in relation to the parenchymal tissue.

In plasma and other tissues the concentration of $^{14}C$ is expressed in d/min per $\mu$l or mg (except for bone marrow) according to the counting efficiency for each tissue.

In plasma (Fig. 3) the disappearance curve of the drug is quite similar in both groups. The curves indicate that the gastrointestinal absorption is fast, with a peak concentration in the plasma occurring between 5 and 15 min. After 6 h, the plasma is almost free of the drug. Except at the 5 min reading, the plasma concentration of $^{14}C$ is always significantly higher in the leukaemic group.

The $^{14}C$ present in the bone marrow from both femurs (Fig. 4), washed thoroughly with a fine needle and a known volume of saline, was measured. Except at the 2 extremities of the curve, the concentration of $^{14}C$ is significantly higher

FIG. 3.—Disappearance curves of radioactivity from the plasma of mice treated with cyclophosphamide-$^{14}C$, 100 mg/kg (about 1 µCi/mouse), orally. Each point represents the mean of 9–10 mice. Vertical bars represent standard errors. Control mice: ●———●, leukaemic mice; ●- - - -●. $^*P < 0{\cdot}05$.

FIG. 4.—Disappearance curves of radioactivity from the bone marrow of mice treated with cyclophosphamide-$^{14}C$, 100 mg/kg (about 1 µCi/mouse), orally. Each point represents the mean of 9–10 mice. Vertical bars represent standard errors. Control mice: ●———●, leukaemic mice: ●- - - -●. $^*P < 0{\cdot}05$.

in the leukaemic group. With a peak concentration at 15 min, the decay of both curves is very slow between the 1st and the 6th h.

In fat taken from the epididymal area (Fig. 5) there is a striking accumulation of $^{14}C$ by the leukaemic mice compared with control mice in which there is a constant concentration up to 60 min, followed by a slow release. Unlike the other tissues, the peak concentration for the leukaemic group is at 30 min, at which interval the $^{14}C$ concentration is 4 times higher than in the control group.

In other tissues (Fig. 6) there is again an overall higher $^{14}C$ concentration in the leukaemic group and where there is not, the differences are not significant. Kidney shows the highest concentration, being the principal site of excretion for cyclophosphamide. Lungs, which are richly vascularized, may reflect the blood concentration, even though some unknown metabolites might be excreted by this route. The liver retains the second highest concentration, followed by the spleen and lungs.

For the gastrointestinal absorption of cyclophosphamide the results are expressed as the percentage of d/min ($^{14}C$) found in the stomach or small intestine, or both. In the content and tissue of the stomach (Fig. 7) we found less $^{14}C$ in the leukaemic group at every point except 60 min, where the findings are not significant. In the same Figure it can be seen that the small intestine (content and tissue) also contains significantly less $^{14}C$ in the leukaemic group, except at 5 and 360 min.

Finally, in Fig. 8 we present the compilation of $^{14}C$ found in the gastrointestinal tract: much less $^{14}C$ is found in the leukaemic group compared with the control group.

### DISCUSSION

There is no doubt, as may be seen from Figs. 3 and 8, that leukaemic mice at an advanced stage of the disease

FIG. 5.—Disappearance curves of radioactivity from the fat of mice treated with cyclophosphamide-$^{14}C$, 100 mg/kg (about 1 μCi/mouse), orally. Each point represents the mean of 9–10 mice. Vertical bars represent standard errors. Control mice: ●———●, leukaemic mice: ●– – – –●. * $P < 0{\cdot}05$.

FIG. 6.—Disappearance curves of radioactivity from lungs, spleen, liver and kidney of mice treated with cyclophosphamide-$^{14}C$, 100 mg/kg (about 1 $\mu$Ci/mouse), orally. Each point represents the mean of 9–10 mice. Vertical bars represent standard errors. Control mice: ●———●, leukaemic mice: ●- - - -●. * $P < 0 \cdot 05$.

show an increased gastrointestinal absorption of cyclophosphamide. The correlation between plasma concentration and the percentage of the administered dose which is found to be present in the gastrointestinal tract indicates that the leukaemic state influences the absorption of cyclophosphamide in $BDF_1$ mice.

In a study of the *in vivo* metabolism of cyclophosphamide, such as the present one, it is of prime importance to have strict control of the evolution of the disease. First of all, the samplings and dosages have been made on the 6th day following the inoculation of $10^6$ leukaemic cells. The median survival time of those mice was 7·7 days (Fig. 1) so that at the 6th day the mice were at the terminal stage. The reddish colour, together with the quantity of ascitic fluid (2–3 ml), constituted 2 additional criteria confirming the evolution of the disease. By the 4th day ascitic fluid was present in the peritoneal cavity and it was very likely the main cause for the increase in body weight (Fig. 2) in leukaemic mice. To avoid misinterpretations of dosages between leukaemic and control mice, cyclophosphamide was administered on the basis of body weight at Day 0, that is on the day of inoculation; at that time, all mice had a weight of 20–23 g. Due to the invasion by leukaemic cells of reticuloendothelial organs such as spleen and liver, the regular observation of a very significant hepatosplenomegaly by

FIG. 7.—Percentage of radioactivity recovered in stomach or small intestine, from 5 to 360 min following an oral dose of cyclophosphamide-$^{14}C$, 100 mg/kg (about 1 $\mu$Ci/mouse). Each point represents the mean of 9–10 mice. Vertical bars represent standard errors. Control mice: ●———●, leukaemic mice: ●– – – –●. * $P < 0 \cdot 05$.

the 6th day (Table I) came as no surprise. In addition, the liver had a markedly pale appearance on section and was very friable.

As far as the plasma disappearance curve is concerned, the results are comparable with those obtained using different methods of adm̃inistration: in newborn mice, the clearance of radioactivity after subcutaneous administration was first order with a half-life of 8·76 h (Bus *et al.*, 1973). In normal mice the clearance of alkylating activity from the blood after a 180 mg/kg intraperitoneal dose was virtually complete within 3 h and alkylating metabolites reached peak plasma concentration at 10–15 min (Gibson and Becker, 1968; Field *et al.*, 1972).

In the bone marrow sampled from the 2 femurs (Fig. 4) an interesting finding was the significant increase in radioactivity, except at 5 min, in leukaemic mice. This could not be simply a reflexion of plasma concentration since an almost constant level of $^{14}C$ has been found between 60 and 360 min, a fact that seems to indicate a selective concentration of the drug at its main site of cytotoxical action (Greenwald, 1973).

The strong increase in radioactivity in the fat tissues of the leukaemic mice (Fig. 5) should be stressed. It is 2–4 times that of the control mice. The fact might be explained in 2 ways: either leukaemic mice have an increased capacity for storing the drug in their fat tissues, due to mechanisms presently unknown, or there might be a decreased biotransformation of cyclophosphamide in the liver so that the drug itself would con-

FIG. 8.—Percentage of radioactivity recovered in the gastrointestinal tract (stomach + small intestine), from 5 to 360 min following an oral dose of cyclophosphamide-$^{14}C$, 100 mg/kg (about 1 $\mu$Ci/mouse). Each point represents the mean of 9–10 mice. Vertical bars represent standard errors. Control mice: ●———●, leukaemic mice: ●- - - -●. * $P < 0{\cdot}05$.

centrate in the fat tissues, *in lieu* of its own metabolites. As demonstrated by several authors, the liver drug metabolizing enzyme activity is reduced in tumour bearing animals, leading to increased levels of unchanged drug and increased pharmacological effect or toxicity (Kato *et al.*, 1968; Kato, Takanaka and Oshima, 1968; Rosso, Dolfini and Donelli, 1968; Rosso *et al.*, 1971; Bartosek *et al.*, 1975; Beck, Mandel and Fabro, 1971, 1975). Such a comparison between control and leukaemic mice is interesting because until now unusually high localization of cyclophosphamide in a specific organ has not been reported (Torkelson, LaBudde and Weikel, 1974).

The distribution of the drug in the other tissues seems to reflect what was observed in plasma, that is a regular and significantly increased concentration of $^{14}C$ in leukaemic mice compared with control mice (Fig. 6). Whereas the plasma disappearance curve had indicated a rapid absorption of the drug with a peak at 15 min, the concentration of $^{14}C$ in the kidneys, which constitute the principal excretory pathway for cyclophosphamide in several animal species (Torkelson *et al.*, 1974) as in man (Bagley, Bostick and DeVita, 1973), would seem to indicate a rapid elimination. However, it had been noted that in mice (Torkelson *et al.*, 1974) as much as 20% of the radioactivity was excreted as $^{14}CO_2$, a fact that would explain the concentrations of $^{14}C$ that have been found in the lungs. The high concentration in the liver can be explained by the important role played by this organ in the biotransformation of cyclophosphamide into cytotoxic alkylating metabolites (Brock and Hohorst, 1962, 1967; Cohen and Jao, 1970; Brock *et al.*, 1971; Sladek, 1972). In mice, the initial oxidative step in cyclophosphamide metabolism is performed by the microsomal mixed function oxidase system of the liver to yield 4-hydroxy cyclophosphamide (Sladek, 1972) which is converted to aldophosphamide; aldophosphamide is very toxic to L-1210 leukaemia cells (Hill *et al.*, 1972) and is further oxidized, probably by liver aldehyde oxidase, to carboxyphosphamide, a metabolite which has little or no anti-tumour effect on L-1210 cells (Hill *et al.*, 1972). More recently, volatile metabolites, possibly produced within the tumour cell but not in the liver, have been isolated, *e.g.* acrolein (Thomson and Colvin, 1974) and phosphoramide mustard (Connors *et al.*, 1974); these 2 metabolites are toxic to L-1210 leukaemia cells and Walker tumour cells. From these studies it appears that the complex mode of activation of cyclophosphamide is not completely resolved (Chabner *et al.*, 1975) and that at least 2 of the known active metabolites (aldophosphamide and acrolein) are indeed labelled with the ring 5-$^{14}$C material used in our study (see Fig. 8 in Chabner *et al.*, 1975).

In the past few years it has been demonstrated that Ehrlich ascites tumour bearing mice (Beck *et al.*, 1975) or spontaneous tumour bearing mice (Sharma and Garb, 1974) as well as Walker 256 carcinoma bearing rats (Rosso *et al.*, 1968; Franchi and Rosso, 1969) cleared drugs (pentobarbitone or zoxazolamine) more slowly than did the normal animals. According to our findings with cyclophosphamide, the increased plasma and tissue radioactivity concentration in leukaemic mice could be explained by an increase in the absorption of cyclophosphamide at the gastric level as well as at the small intestine level (Fig. 7) (see Fig. 8 for a summary). The hypothesis might be advanced that an increase in gastrointestinal motility would facilitate the transit of the drug and accelerate its passive diffusion to the blood. There may also be an increased blood flow at the level of the gastrointestinal tract in leukaemic mice. The actual mechanism remains to be demonstrated. We may add, from the data appearing in Table II, that a definite correlation can hardly be made between the degree of tissue infiltration by malignant cells and drug uptake by the same tissues.

It is important to note that the experiments were also performed on leukaemic mice 4 days after the inoculation of $10^6$ leukaemic cells and that, at that stage, there was no difference between leukaemic and control mice as far as the metabolism (gastrointestinal absorption, plasma and tissue concentration) of cyclophosphamide was concerned.

In conclusion, our findings confirm the importance of a pharmacokinetic approach in the study of antineoplastic drugs (Balconi *et al.*, 1973; Bischoff, 1973; Mellet, 1974; Bossi *et al.*, 1975) even if it is well known that the efficacy of cyclophosphamide is dependent on its biotransformation by the liver (Sladek 1972). Leukaemic mice at an advanced stage of the disease do not metabolize cyclophosphamide as the controls, or as they themselves do at an earlier stage of their disease. Such findings, if they can be extrapolated to man, would indicate the need for an adequate control of cyclophosphamide administration, particularly with regard to avoiding excess concentrations leading to intolerable side-effects.

This investigation was supported by the Medical Research Council of Quebec (Grant No. 740076) and by the Medical Research Council of Canada (Grant No. MA-5561).

### REFERENCES

BAGLEY, C. M., BOSTICK, F. W. & DEVITA, V. T. (1973) Clinical Pharmacology of Cyclophosphamide. *Cancer Res.*, **33**, 226.

BALCONI, G., BOSSI, A., DONELLI, M. G., FILIPPESCHI, S., FRANCHI, G., MORASCA, L. & GARATTINI, S. (1973) Chemotherapy of a Spontaneous Mammary Carcinoma in Mice: Relation between *in vitro–in vivo* Activity and Blood and Tumor Concentrations of Several Antitumor Drugs. *Cancer Chemother. Rep.*, **57**, 115.

BARTOSEK, I., DONELLI, M. G., GUAITANI, A., COLOMBO, T., ROSSO, R. & GARATTINI, S. (1975) Differences of Cyclophosphamide and 6-mercaptopurine Metabolic Rates in Perfused Liver of Normal and Tumour-bearing Animals. *Biochem. Pharmac.*, **24**, 289.

BECK, W. T., MANDEL, H. G. & FABRO, S. (1971) Increased Sleeping Time in Mice Bearing the Ehrlich Ascites Tumor. *Fedn Proc.*, **30**, 398.

BECK, W. T., MANDEL, H. G. & FABRO, S. (1975) Physiological Disposition of Pentobarbital in Tumor-bearing Mice. *Cancer Res.*, **35**, 1333.

BISCHOFF, K. B. (1973) Pharmacokinetics and Cancer Chemotherapy. *J. pharmacokin. Biopharm.*, **1**, 465.

BOSSI, A., COLOMBO, T., DONELLI, M. G. & GARATTINI, S. (1975) Activity of Cyclophosphamide and 1-methylnitrosourea on Ehrlich Carcinoma Transplanted in Different Sites. Correlation between Drug Level and Tumour Inhibition. *Biochem. Pharmac.*, **24**, 21.

BROCK, N. & HOHORST, H. J. (1962) Uber die Aktivierung von Cyklophosphamid in Wärmblüterorganismus. *Naturwissenschaften*, **49**, 610.

BROCK, N. & HOHORST, H. J. (1967) Metabolism of Cyclophosphamide. *Cancer, N.Y.*, **20**, 900.

BROCK, N., GROSS, R., HOHORST, H. J., KLEIN, H. O. & SCHNEIDER, B. (1971) Activation of Cyclophosphamide in Man and Animals. *Cancer, N.Y.*, **27**, 1512.

BUS, J. S., SHORT, R. D. & GIBSON, J. E. (1973) Effect of Phenobarbital and SKF 525-A on the Toxicity, Elimination and Metabolism of Cyclophosphamide in Newborn Mice. *J. Pharm. exp. Ther.*, **184**, 749.

CHABNER, B. A., MYERS, C. E., COLEMAN, C. N. & JOHNS, D. G. (1975) The Clinical Pharmacology of Antineoplastic Agents (First of Two Parts). *New Engl. J. Med.*, **292**, 1107.

COHEN, J. L. & JAO, J. Y. (1970) Enzymatic Basis of Cyclophosphamide Activation by Hepatic Microsomes of the Rat. *J. Pharm. exp. Ther.*, **174**, 206.

CONNORS, T. A., COX, P. J., FARMER, P. B., FOSTER, A. B. & JARMAN, M. (1974) Some Studies of the Active Intermediates Formed in the Microsomal Metabolism of Cyclophosphamide and Isophosphamide. *Biochem. Pharmac.*, **23**, 115.

FIELD, R. B., GANG, M., KLINE, I., VENDITTI, J. M. & WARAVDEKAR, V. S. (1972) The Effect of Phenobarbital or 2-diethylaminoethyl-2,2-diphenylvalerate on the Activation of Cyclophosphamide *in vivo*. *J. Pharm. exp. Ther.*, **180**, 475.

FRANCHI, G. & ROSSO, R. (1969) Metabolic Fate of Zoxazolamine in Tumor-bearing Rats. *Biochem. Pharmac.*, **18**, 236.

FRIEDMAN, O. M. & BOGER, E. (1961) Colorimetric Estimation of Nitrogen Mustards in Aqueous Media. *Analyt. Chem.*, **33**, 906.

GERSHWIN, M. E., GOETZL, E. J. & STEINBERG, A. D. (1974) Cyclophosphamide: Use in Practice. *Ann. intern. Med.*, **80**, 531.

GIBSON, J. E. & BECKER, B. A. (1968) Effect of Phenobarbital and SKF 525-A on the Teratogenicity of Cyclophosphamide in Mice. *Teratology*, **1**, 393.

GREENWALD, E. S. (1973) Alkylating Agents. In *Cancer Chemotherapy*. New-York: Med Examination Publishing Co Inc.

HILL, D. L., LASTER, W. R. JR & STRUCK, R. F. (1972) Enzymatic Metabolism of Cyclophosphamide and Nicotine and Production of a Toxic Cyclophosphamide Metabolite. *Cancer Res.*, **32**, 658.

KATO, R., TAKANAKA, A. & OSHIMA, T. (1968) Drug Metabolism in Tumor-bearing Rats. II. *In vivo* Metabolisms and Effects of Drugs in Tumor-bearing Rats. *Jap. J. Pharmac.*, **18**, 245.

KATO, R., TAKANAKA, A., TAKAHASHI, A. & ONADA, K. (1968) Drug Metabolism in Tumor-bearing Rats. I. Activities of NADPH-linked Electron Transport and Drug-metabolizing Systems in Liver Microsomes of Tumor-bearing Rats. *Jap. J. Pharmac.*, **18**, 224.

MELLET, L. B. (1974) Pharmacodynamic and Pharmacokinetic Measurements of Antitumor Agents. *Clin. pharmac. Ther.*, **16**, 230.

ROSSO, R., DOLFINI, E. & DONELLI, M. G. (1968) Prolonged Effect of Pentobarbital in Tumor-bearing Rats. *Eur. J. Cancer*, **4**, 133.

ROSSO, R., DONELLI, M. G., FRANCHI, G. & GARATTINI, S. (1971) Impairment of Drug Metabolism in Tumor-bearing Animals. *Eur. J. Cancer*, **7**, 565.

SHARMA, G. C. & GARB, S. (1974) Influence of Cancer on the Levels of Pentobarbital in the Blood and Brain of Mice. *Gann*, **65**, 467.

SLADEK, N. E. (1972) Therapeutic Efficacy of Cyclophosphamide as a Function of its Metabolism. *Cancer Res.*, **32**, 535.

THOMSON, M. & COLVIN, M. (1974) Chemical Oxidation of Cyclophosphamide and 4-methylcyclophosphamide. *Cancer Res.*, **34**, 981.

TORKELSON, A. R., LABUDDE, J. A. & WEIKEL, J. H. JR (1974) The Metabolic Fate of Cyclophosphamide. *Drug. Metab. Rev.*, **3**, 131.